# The Effect of Topical Substance-P Plus Insulin-like Growth Factor-1 (IGF-1) on Epithelial Healing After Photorefractive Keratectomy in Rabbits

**DOI:** 10.1167/tvst.7.1.12

**Published:** 2018-01-23

**Authors:** Zahra Ghiasi, Tracy Gray, Phat Tran, Richard Dubielzig, Chris Murphy, David L. McCartney, Ted W. Reid

**Affiliations:** 1 Department of Ophthalmology and Visual Sciences, Texas Tech University Health Science Center, Lubbock, TX, USA; 2 Department of Surgical Services, School of Veterinary Medicine, University of Wisconsin, Madison, WI, USA; 3 Department of Veterinary Surgical and Radiological Sciences, University of California, Davis, CA, USA

**Keywords:** Substance P, IGF-1, wound healing, photorefractive keratectomy

## Abstract

**Purpose:**

To determine whether topical Substance-P (SP) plus insulin-like growth factor-1 (IGF-1) can improve corneal healing after photorefractive surface ablation in a rabbit.

**Methods:**

After a 9.0-mm corneal de-epithelialization using a combination of chemical (18% alcohol) and mechanical debridement, excimer photorefractive surface ablation was performed bilaterally in eight rabbits (16 eyes) with an 8.0-mm ablation zone and 70-μm depth. The right eye was treated with SP (250 μg/mL) and IGF-1 (25 ng/mL) in hyaluronic acid, one drop twice a day, and the other eye treated with only hyaluronic acid. The epithelial healing process was documented photographically twice a day until healing was complete. Six rabbits were sacrificed 6 weeks after photorefractive keratectomy (PRK) and corneas examined histologically.

**Results:**

Seven of eight rabbit eyes treated with SP/IGF-1 healed in a shorter time than the untreated eye. For rabbit #6, both eyes healed at the same time. The average healing time (total time until wound closure) for the treated eyes was 99 hours, while the average healing time for the untreated eyes was 170 hours (*P* = 0.0490). A persistent epithelial defect was found in two of the nontreated eyes but none in the treated eyes. Corneal pathology showed some degree of epithelial separation in the central corneal wound in three out of six nontreated eyes and in just the treated eye of rabbit #6.

**Conclusion:**

Topical SP plus IGF-1 increases the epithelial healing rate after PRK. There may have been beneficial effects upon cell adhesion as well.

**Translational Relevance:**

Better and faster healing.

## Introduction

Removal of the corneal epithelium is necessary before photorefractive keratectomy (PRK). Corneal wound healing after PRK and its consequences, epithelial defect and haze, has been studied extensively. Results on how the absence of corneal epithelium affects the stromal keratocytes are contradictory. Some studies showed that an early decrease in the density of keratocytes is followed by an increased number of these cells in the underlying stroma and polymorphonuclear (PMN) inflammatory reaction.^1,2^ These stromal changes are related to stromal haze and stability of the refractive result. Other studies indicate that an atraumatic removal of the epithelium would prevent changes in the stromal healing.^3,4^

Studies suggest that the epithelium influences the cellular activation and metabolic activity of stromal cells during wound healing.^5,6^ Removal of the corneal epithelium causes loss of the superficial stromal keratocytes in rabbits and monkeys. This keratocyte death may result from osmotic changes that alter the corneal healing process.^7^ The use of corneal preservation medium as a nutrient solution during and immediately after de-epithelialization of rabbit corneas resulted in healthy superficial keratocytes and faster re-epithelialization.^7^

The presence of PMN leukocytes in the stroma might be related to the regeneration of the epithelial cells^2^ or the cells may be stimulated by chemotactic factors liberated by the degenerating keratocytes. The timing of all these findings suggests an interaction not only between the epithelium and keratocytes but also between epithelium and PMN leukocytes.^2,4^ Clinically, these changes may contribute to melting of the underlying stroma in areas of persistent epithelial defects.

The apparent importance of rapid epithelial healing in improvement of the final results in PRK patients prompted us to evaluate the effect of topical Substance-P (SP) plus insulin-like growth factor-1 (IGF-1) in epithelial healing after PRK in a rabbit eye. SP is an 11-amino acid peptide belonging to the tachykinin family of sensory neurotransmitters and is found in corneal sensory nerves. Reid et al.^8,9^ demonstrated that SP is mitogenic for ocular epithelial cells. In addition, topical administration of SP to rabbit cornea epithelial defects stimulates DNA synthesis and cell growth.^10^ These results were later reviewed in the context of the role of nerves in the cornea.^11^ It was later shown that topical SP could heal corneal defects in rats and dogs.^12,13^ In vitro, it was also found that SP plus IGF-1 would stimulation the migration of epithelial cells onto a bare rabbit corneal stroma while the individual compounds failed to show a beneficial effect.^14^ Later, it was shown that SP in conjunction with IGF-1 stimulated the healing of nonhealing ulcers of a child with familial dysautonomia,^15^ and in a patient with a diabetic ulcer.^16^

## Material and Methods

All experiments were conducted according to the Association for Research in Vision and Ophthalmology Statement for Use of Animals in Ophthalmic and Vision Research. The protocol was approved by the Animal Research committee of the Texas Tech University Health Science Center.

### Excimer Photoablation and Treatment Protocol

Six New Zealand albino female rabbits weighting between 2 and 3 kg were anesthetized with intramuscular injection of xylazine hydrochloride (7 mg/mL) and ketamine hydrochloride (40 mg/mL). Topical 0.5% proparacaine hydrochloride was instilled and the eyelids were held open with a wire speculum. After dilation of the pupils with a drop of 2.5% phenylephrine and 1% tropicamide, the animals underwent a bilateral combined chemical (18% alcohol for 45 seconds) and mechanical corneal de-epithelialization of 9.0 mm in diameter. Both corneas then received 193 nm Excimer laser using a LADARVision 4000 laser with an 8.0-mm ablation zone and a depth of 70.0 μm (−3.00 myopic correction). The beam size was 0.75 mm and the pulse repetition rate was 60 Hz, with an average fluence of 200 mJ per square centimeter.

At the conclusion of the laser treatment, a drop of 2% sodium fluorescein solution was instilled in the conjunctival cul de sacs, and the corneal epithelial defect was assessed and documented by digital photo-documentation. Then one drop of combined SP (250 μg/mL) and IGF-1 (25 ng/mL) in hyaluronic acid vehicle was instilled in the right. The left eye received only eye drops of hyaluronic acid and was used as a control. Hyaluronic acid was used as a vehicle since it was found that the signal from SP should be present for at least 2 hours in order to stimulate mitosis and the hyaluronic acid increased the resident time of SP on the cornea.^9^

### Postoperative Regimen and Follow-up

Postoperatively, one drop of SP (250 μg/mL) and IGF-1 (25 ng/mL) in hyaluronic acid vehicle was instilled in the right eyes twice a day, and the corneal epithelial defect was assessed and documented by digital photo-documentation before each treatment.

For rabbits #7 and 8, after healing of the treated eye, at 180 hours the rabbits were sacrificed for humane reasons since the control eye had not healed. All other rabbits (#1–6) were killed at 6 weeks. The eyes were fixed in Bouin's solution and processed for histology.

### Statistical Analyses

Results were statistically analyzed using GraphPad InStat 3.06 (GraphPad Software, San Diego, CA). Significance between pairs of values (control vs. one treatment group) was calculated using an unpaired two-tailed Student's *t*-test when SD was not significantly different and when a Gaussian distribution was observed. If SD was significantly different, the Welch correction was applied to the unpaired two-tailed Student's *t*-test. When non-Gaussian distribution was observed (Kolmogorov-Smirnov test), significance was calculated by a nonparametric Mann-Whitney *U* test. Differences were considered significant when the *P*-value was greater than 0.1.

## Results

Epithelial defects created by the chemical and mechanical technique healed uneventfully in all eyes treated with SP/IGF-1, with an average healing time of 99 hours. Two of the nontreated eyes (left eye) developed persistent epithelial defects. The average healing time for nontreated eye was 170 hours. The results for the rate of wound healing are available for the eight rabbits (See Fig. 2A-H).

The difference between total healing time in treated and nontreated eyes was significant (*P* = 0.0490). For only one rabbit was the healing time the same (#6).

**Figure 1:**
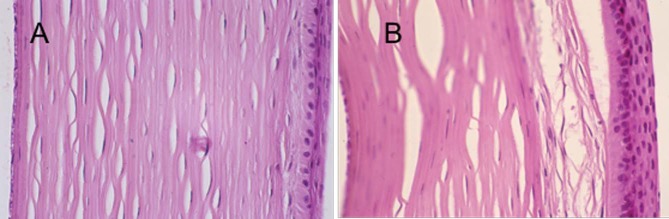
Hematoxylin and eosin histology of rabbit corneal wounds after PRK and healing. (A) Treated with SP/IGF-1 (190×); (B) control (190×).

We also noticed the maximum effect of our treatment regimen was shown around day three (73–80 hours after treatment) with all rabbits except rabbit #8. While the initial healing rate was similar for all the rabbits, in all cases (both treated and control), it can be seen that after this initial healing, the area actually increased for a time in several of the control eyes (Fig. 1).

**Figure 2:**
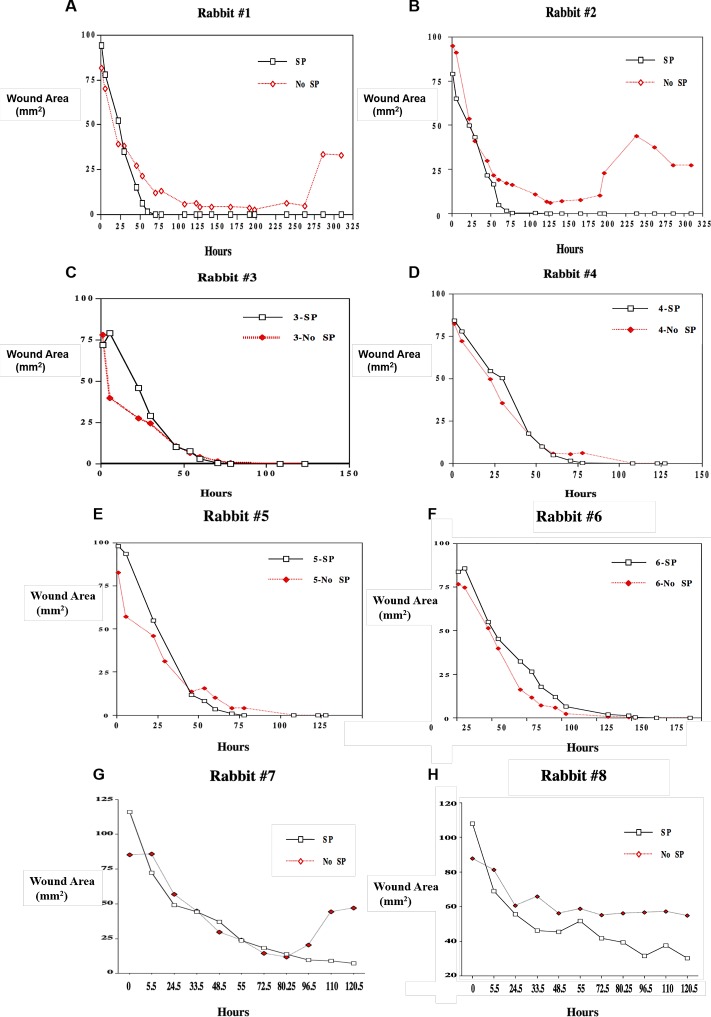
Plot of wound healing results for the rabbit corneas after PRK treatment, wound area (mm^2^) versus hours after treatment (SP/IGF-1, or vehicle only).

### Histology Studies

The six rabbits with healing of both eyes were sacrificed 6 weeks after PRK, and the corneal samples were sectioned for histopathology. Rabbits 7 and 8 were not included because the healing of the untreated eye was not complete. There were two major histology findings, subepithelial pannus of collagen and spindle cells and, epithelial nonattachment. A summary is listed below:

In rabbit #1 there was minimal subepithelial pannus formation and no epithelial separation in both treated and nontreated eyes.

In rabbit #2 there was moderate subepithelial pannus formation in both eyes, but there was an area of epithelial separation in the central cornea in the nontreated eye.

In rabbit #3 there was severe subepithelial pannus formation with no epithelial separation in both eyes. But nontreated eye showed a localized area of thick subepithelial pannus in the central cornea with evidence of atrophy in the overlying epithelium but no epithelial separation.

In rabbit #4 there was moderate subepithelial pannus formation in both eyes with a zone of epithelial separation within the pannus in treated eye and broad epithelial separation in the untreated eye.

In rabbit #5 we noticed minimal subepithelial pannus of collagen and spindle cells and no evidence of epithelial separation in the treated eye, while in the untreated eye severe pannus formation and localized epithelial separation with separated clefts within the pannus were prominent.

And finally, in rabbit #6 there was severe pannus formation and epithelial separation in the treated eye, and moderate pannus formation with no evidence of epithelial separation in the left eye.

In summary, the pathology results showed that in three out of six nontreated eyes there is evidence of epithelial separation. Also, in one nontreated eye there was an area of subepithelial atrophy that can predispose the epithelium for separation. In only one treated eye (#6) did we notice epithelial separation. Representative sections from rabbit #5 are seen in Figure 2.

## Discussion

The wound in a denuded cornea shows an intensive inflammatory response and an absence of keratocytes.^7,17,18^ The phenomenon of epithelial-loss-induced keratocyte loss has been characterized as a process of apoptosis^19^ and may be mediated by interleukin-1 (IL-1) elaborated by epithelial injury,^20^ and reactive oxygen free radicals generated by excimer laser irradiation,^21^ and acute inflammatory cells. In addition, this keratocyte death may result from osmotic and metabolic changes of stromal cells related to epithelial denuation.^7^

The presence of PMN leukocytes in the stroma might be related to the regeneration of the epithelial cells^2^ or by chemotactic factors liberated by the degenerating keratocytes. The timing of all these findings suggests an interaction not only between the epithelium and keratocytes but also between epithelium and PMN leukocytes.^2,4^ Clinically, this may contribute to melting of the underlying stroma in the persistent epithelial defects.

The above changes may cause photoablated corneal wounds to undergo a transitional stage of epithelial hyperplasia and stromal keratocytes proliferation, which in turn leads to corneal scaring and haze in a later stage.^22^

The cornea is more densely innervated with sensory nerve fibers than any other tissue in the body. There is statistically significant less hemidesmosome formation up to 12 weeks in corneas after mechanical de-epithelialization with or without PRK.^23^ This will affect epithelial adhesion complex and corneal barrier function.^23,24^ Additional changes after PRK may be due to sensory denervation of the cornea following the treatment, which can affect epithelial healing and cellular adhesion.^25,26^

In the cornea, a dense network of SP positive nerve fibers has been reported.^27,28^ SP is a constituent of sensory nerve fibers and has been postulated to mediate various physiologic functions.^29^ It has a key role in the ocular neurogenic responses to various stimuli.^28,30^ The SP level in the cornea of adult mouse is reduced 60% by denervation of the trigeminal nerve.^31^ The receptor for SP has been reported in corneal epithelial cells.^31,32^

**Figure 3:**
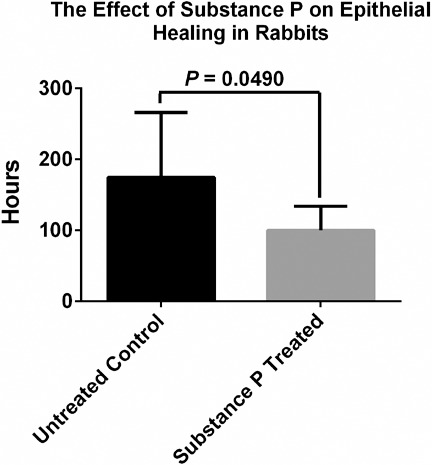
Average total healing times after PRK treatment, for eyes treated with SP/IGF-1 or untreated controls.

While SP or IGF-1 alone does not influence the migration of the corneal epithelial cells, it has been shown that there is a synergistic effect between SP and IGF-1 in corneal epithelial migration (Fig. 3).^14^ This synergistic effect can be nulled by the addition of an SP receptor antagonist. This indicates that the synergistic effect of SP with IGF-1 is mediated by SP receptors in the corneal epithelium.^14^

A continuous renewal of epithelial cells through the active repair system is one of the most important mechanisms for the maintenance of epithelial integrity. During this process, migration and cell adhesion are the most important processes.^34^ There are three phases involved in the process of corneal healing: (1) migration of corneal epithelial cells forming a monolayer of epithelial cells, (2) proliferation of the monolayer epithelial cells, and (3) differentiation to the different epithelial cell layers.^35^

The synergistic effect of SP and IGF-1 has been reported for both epithelial migration and attachment during the first phase of epithelial healing. Treatment of the corneal epithelial cells with SP and IGF-1 stimulates the attachment of cells to various extracellular material proteins.^36^ The mechanism of action for SP+IGF-1 might be mediated by the upregulation of fibronectin receptor in the corneal epithelial cells.^14,36^ In addition, SP also stimulates DNA synthesis in rabbit corneal epithelial cells.^9^

More recently it was shown that SP promotes diabetic corneal epithelial wound healing in mice through the neurokinin-1 (NK-1) receptor.^37^ They found that the NK-1 receptor contributes to the promotion of diabetic corneal epithelial wound healing by rescued activation of RAC-alpha serine/threonine-protein kinase (Akt), epidermal growth factor receptor (EGFR), and sirtuin 1 (Sirt1) improvement of mitochondrial function, and increased reactive oxygen species scavenging capacity.^38^ They also showed that SP enhances keratocyte migration and neutrophil recruitment through IL-8.^38^ These findings point to the critical role that SP plays in the wound healing process and the importance of corneal nerves.

As mentioned before, the cornea is heavily innervated with sensory nerve fibers, and this innervation plays an important role in the maintenance of normal structure and function of the cornea. Several authors have demonstrated a correlation between the reduced SP level in the cornea and denervation of the trigeminal nerve to the eye.^31,39,40^

These clinical observations and laboratory studies strengthen our understanding that neuropeptides such as SP play an important role in the physiology of corneal epithelial cells and their healing processes. As we observed in our study, the main difference in healing rate between treated and nontreated eyes occurred 70 to 90 hours after PRK. This timing correlates with the time after the initial movement of epithelial cells onto the denuded area when they began to proliferate and attach. Based upon our previous studies of SP+IGF, we expect these factors to have a significant effect on epithelial migration, attachment, and proliferation. We think this effect of SP+IGF is seen in the faster completion of the wound closure process for the SP/IGF-1 treated eyes (99 vs. 170 hours). This is also consistent with the fact that in some cases the untreated wounds showed an increase in wound area during the healing process. This may be due to incomplete attachment of the epithelium, due to a lack of SP, resulting from the obliteration of the sensory nerve endings during the excimer ablation treatment.

## Conclusions

These results suggest that SP/IGF-1 plays a significant role in wound healing of corneal wounds and may be useful in improving the outcome after excimer surface ablation surgery.
